# The Triumphs of Electricity

**Published:** 1896-03

**Authors:** 


### The Triumphs of Electricity.

The triumphs of electricity in the domain of practical science exceed all expectation. It is converting clay into aluminum, by Hall's process, at so small a cost that it promises to supersede all other metals in the industrial arts. An electrician, in Tennessee, has taken out a patent for the destruction of weeds along railway lines by electricity. This seems to foreshadow its use for a like purpose in agriculture. A Milwaukee inventor burns bricks in three hours and a half by means of an electric current. In France some of the tanneries are already completing by the electric method in four days the tanning process which used to occupy eighteen months; and in both France and England electricity is being successfully employed in purifying sewage and generating ozone, while a medical practitioner, of high repute in the United States, does not hesitate to declare that " whenever medical electricity shall be generally adopted in practice, ulcers and abscesses will be as rare as comets."—*Indian Lancet.*

